# Research Progress on Nasal Delivery of siRNA Nanocarrier Systems for the Treatment of Neurodegenerative Diseases

**DOI:** 10.3390/pharmaceutics17111407

**Published:** 2025-10-30

**Authors:** Qingqing Huang, Wei Wu, Yinghai Liu, Weiqing Li, Xin Chen, Sixun Yu, Gu Gong, Haifeng Shu

**Affiliations:** 1 Department of Neurosurgery, The General Hospital of Western Theater Command, College of Medicine, Southwest Jiaotong University, Chengdu 610083, China; corn1993@my.swjtu.edu.cn (Q.H.); chenxin0852@hotmail.com (X.C.); ysx1982@aliyun.com (S.Y.); 2 Department of Anesthesiology, The General Hospital of Western Theater Command, Chengdu 610083, China; wuwei731@outlook.com (W.W.); liuyinghai1978@outlook.com (Y.L.);; 3 Tissue Stress Injury and Functional Repair Key Laboratory of Sichuan Province, Chengdu 610083, China

**Keywords:** nasal delivery, siRNA, nanocarriers, central nervous system diseases, blood-brain barrier, dendritic polymers, non-invasive delivery

## Abstract

The treatment of central nervous system (CNS) diseases faces huge challenges, mainly due to the blood–brain barrier (BBB) restricting drug delivery, which leads to many potential treatment methods being unable to effectively reach the target area. In recent years, nasal administration has received extensive attention as a non-invasive drug delivery route because of its anatomical connection with the brain, enabling direct delivery to brain tissue. In particular, the siRNA delivery system based on nanocarriers has shown great promise in the treatment of CNS diseases due to its unique advantages in targeting gene silencing. This article reviews the latest research progress on nasal administration of siRNA nanocarriers, with a focus on the design strategies, administration mechanisms, in vivo and in vitro effects, and safety evaluations of different nanocarriers. The aim is to provide a systematic theoretical basis and future research directions for the application of siRNA nasal administration in the treatment of CNS diseases (see the abstract of the picture).

## 1. Introduction

The treatment of central nervous system (CNS) diseases is an important challenge in medical research. Among them, the major physiological barrier formed by the blood–brain barrier (BBB) limits the effective entry of therapeutic drugs into the brain parenchyma, significantly reducing the efficacy of drug therapy [1,2]. Many candidate drugs perform poorly in the clinical research phase targeting CNS diseases, often due to their inability to effectively cross the BBB and reach the required therapeutic concentration at the target site [3,4]. Structurally, the BBB is mainly composed of brain microvascular endothelial cells sealed by tight junctions, supported by pericytes, astrocytic end-feet, and the basement membrane, together forming a neurovascular unit. This complex organization maintains CNS homeostasis but also poses a formidable obstacle for therapeutic agents, including siRNA, to reach brain parenchyma [5]. Although there are invasive administration methods, such as intrathecal injection, parenchymal or intraventricular injection, which can directly deliver the drug to the brain, these methods themselves carry higher risks and have lower patient compliance [6,7].

To overcome this delivery bottleneck, non-invasive delivery strategies have received widespread attention. Intranasal delivery has shown unique advantages as it can bypass BBB through the olfactory and trigeminal nerve pathways, delivering drugs directly to the cerebrospinal fluid (CSF) and brain tissue [8,9]. This route not only avoids the limitations of the BBB but also enables rapid and effective drug delivery, significantly increasing the bioavailability of drugs in the CSF [1,10]. Studies have shown that intranasal delivery can efficiently deliver drugs into the brain in a non-invasive manner, reducing the burden on patients while also lowering the risk of systemic side effects [11,12].

Small interfering RNA (siRNA) as a powerful gene silencing tool has great potential in targeted therapy for CNS diseases. However, its clinical translation faces core challenges such as poor stability and low delivery efficiency [13,14]. Nanocarrier systems, particularly dendrimers, have shown exceptional potential in enhancing siRNA delivery efficiency and targeting. These carriers not only protect siRNA from nuclease degradation but also significantly improve its membrane translocation ability, thereby enhancing its bioactivity within target cells [13,15].

This article aims to systematically review the current application status and core challenges of siRNA nanocarrier systems based on the nasal administration route in the treatment of CNS diseases, and to explore its potential and application prospects in enhancing delivery efficiency, reducing adverse effects, and improving treatment experience. Combining the latest research progress, this article hopes to provide new ideas for developing more effective treatment strategies for CNS diseases.

## 2. Research Background of CNS Diseases and siRNA Therapy

### 2.1. Pathologic Mechanisms and Treatment Difficulties of CNS Diseases

The pathological mechanisms of CNS diseases are complex and diverse, involving various factors such as abnormal protein expression and neuroinflammatory responses. Taking Alzheimer’s disease (AD) and Parkinson’s disease (PD) as examples, the pathogenesis of these two types of neurodegenerative diseases is closely related to the accumulation of abnormal proteins [16,17]. In AD, the deposition of amyloid beta (Aβ) leads to neuronal damage, which in turn causes cognitive impairment [16]; while in PD, the aggregation of alpha-synuclein forms Lewy bodies that can mediate the death of dopaminergic neurons [17]. These abnormal proteins not only directly damage nerve cells but can also exacerbate neuronal degeneration by activating neuroinflammatory responses, creating a vicious cycle [16,17].

Currently, the treatment of CNS diseases faces significant challenges. Traditional drug therapies often struggle to achieve effective concentrations at the lesion site due to the limitations of the BBB, resulting in restricted efficacy. For example, the efficacy of AD treatment drugs in clinical trials has been poor, partly attributed to the low penetration efficiency of the BBB [18]. Additionally, the complex interactions among tumor cells, immune cells, and stromal cells in the brain tumor microenvironment, as well as the pathological microenvironment of neuroinflammatory diseases, further increase the difficulty of treatment [19]. Neuroinflammation is one of the key challenges in the treatment of CNS diseases. Chronic inflammation not only accelerates neuronal degeneration but may also expand the range of neuronal injury. In multiple sclerosis (MS), abnormal activation of microglia can promote neuronal damage and demyelination processes [20], indicating a direct correlation between neuroinflammation and disease progression. Therefore, it is crucial to develop novel therapeutic strategies targeting neuroinflammation regulation.

In summary, CNS diseases involve a complex network of various cell types and their interactions, and the limitations of traditional drug therapies need to be overcome urgently. Novel delivery strategies based on siRNA nanocarrier systems are expected to provide new ideas for improving the prognosis of CNS diseases by efficiently penetrating the BBB and precisely regulating target gene expression.

### 2.2. The Mechanism of Action of siRNA in CNS Diseases

siRNA exerts its therapeutic effects by harnessing the RNA interference (RNAi) pathway, in which complementary binding between siRNA and target mRNA leads to selective degradation of the transcript and subsequent gene silencing [21]. This mechanism allows siRNA to intervene in multiple shared pathogenic processes of CNS diseases, such as abnormal gene expression, protein misfolding, and neuroinflammation, while also enabling disease-specific interventions.

In AD, siRNA targeting ApoE or APP reduces amyloid-beta deposition and promotes neuronal protection [22,23]. In Huntington’s disease (HD), silencing of HTT or MSH3 genes mitigates mutant protein toxicity and slows disease progression [21]. siRNA has also shown therapeutic potential in PD, where inhibition of α-synuclein (SNCA) expression decreases protein aggregation and preserves dopaminergic neurons, thereby alleviating motor deficits [24]. Similarly, in amyotrophic lateral sclerosis (ALS), siRNA against SOD1 delays neurodegeneration and prolongs survival in transgenic mouse models [25]. siRNA can further modulate neuroinflammation, for example, by targeting TNF-α, reducing inflammatory injury and improving neural outcomes [26]. In epilepsy, siRNA strategies directed against pro-excitatory mediators such as NMDAR subunits or IL-1β have been shown to attenuate excitotoxicity and seizure severity [27]. Beyond neurodegenerative and inflammatory disorders, siRNA has also been applied in brain tumors, where silencing of oncogenes suppresses proliferation and migration of malignant cells [28]. Collectively, these findings demonstrate that while CNS diseases differ in their clinical manifestations, they share common molecular mechanisms—such as protein aggregation, inflammatory cascades, and aberrant gene expression—that can be effectively targeted by siRNA.

Despite these advances, clinical application remains challenging. siRNA is rapidly degraded in vivo with a short half-life [29], and cellular uptake efficiency is low. Although nanocarriers such as liposomes have improved stability and delivery [30], the BBB (BBB) remains a formidable obstacle. To address these issues, innovative strategies, particularly intranasal administration, are being actively investigated to enhance brain targeting and therapeutic efficacy [9].

## 3. The Anatomical Basis and Advantages of the Nasal Cavity Delivery System

### 3.1. The Anatomical Connection Between the Nasal Cavity and the Brain

**Figure 1:**
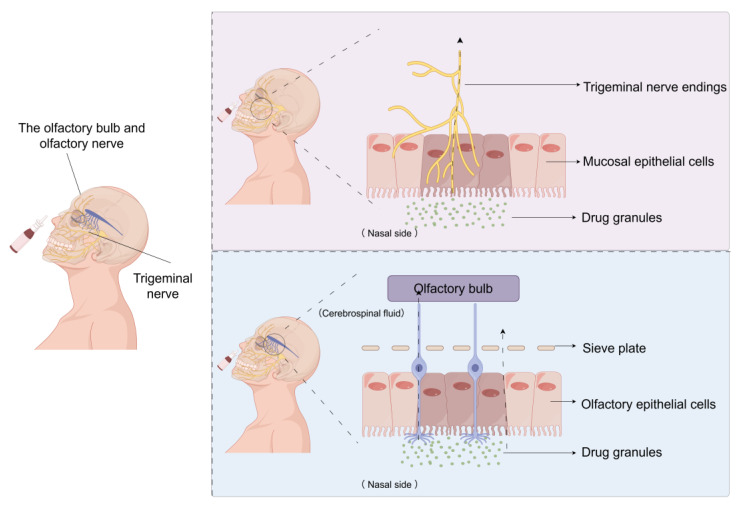
Schematic illustration of nasal-to-brain anatomical pathways. Intranasally administered siRNA nanocarriers can be transported to the brain via two major anatomical routes: (i) the olfactory pathway, where carriers cross the olfactory epithelium and reach the olfactory bulb, and (ii) the trigeminal pathway, where carriers penetrate the respiratory epithelium and are transported through trigeminal nerve branches. These pathways bypass the BBB and allow direct access to CNS tissue. Abbreviations: BBB, blood–brain barrier; CNS, central nervous system. [Created by Figdraw (www.figdraw.com, Accessed on: 12 September 2025), ID: ATROIdfdbb].

As illustrated in Figure 1, the anatomical connection between the nasal cavity and the brain mainly relies on the pathways of the olfactory nerve and the trigeminal nerve. Among them, the olfactory nerve, as the core pathway directly connecting the nasal cavity to the olfactory bulb of the brain, constitutes the main route for drugs administered intranasally to reach the brain, providing a unique advantage for the treatment of CNS diseases. Meanwhile, the trigeminal nerve also plays an important role, as its branches distributed in the nasal cavity participate in facial sensory conduction, further enhancing the delivery efficiency of drugs from the nasal cavity to the brain [31].

From a local anatomical structure perspective, the inherent spatial structure of the nasal cavity creates favorable conditions for drug deposition. The rich vascular network and olfactory epithelium not only significantly enhance drug absorption efficiency but also optimize its bioavailability [32,33]. In addition, the study of individual differences in the nasal cavity’s anatomical structure is crucial for optimizing drug delivery systems. These characteristics collectively contribute to the efficient deposition of drugs in the nasal cavity, thereby enhancing their targeting and therapeutic effects on the CNS [32,33].

In terms of traditional bottlenecks, the BBB has long restricted the delivery of drugs to the CNS [34]. The breakthrough of nasal administration lies in achieving direct nose-to-brain drug transport through the olfactory and trigeminal pathways, significantly shortening the time for drugs to reach the target point and improving therapeutic efficiency [34,35]. It is worth emphasizing that this non-invasive administration method has good patient tolerance, allowing drugs to rapidly distribute to brain tissue within minutes, effectively avoiding the first-pass effect in the liver and systemic side effects [36,37]. This characteristic demonstrates significant advantages in the treatment of CNS diseases that require rapid onset, such as seizures and AD [36,37].

In summary, the anatomical characteristics of the nasal–brain pathway lay a solid foundation for nasal drug delivery. Future research directions should include further enhancing bioavailability through innovations in drug formulation and optimization of delivery technologies, while deepening the study of nasal physiological characteristics to provide theoretical support for the development of a new generation of CNS-targeted therapies.

### 3.2. Advantages and Potential Limitations of Nasal Delivery

The nasal delivery system has attracted attention in the treatment of CNS diseases due to its non-invasive characteristics. Compared to traditional injections or surgical methods, this approach provides patients with a more convenient and friendly treatment option. This method of drug administration can deliver drugs directly to the brain through the olfactory and CSF pathways, providing a non-invasive alternative to systemic delivery, thereby significantly enhancing the brain bioavailability and therapeutic effect of the drugs [38]. Existing clinical studies have confirmed that nasal delivery can effectively alleviate patients’ pain and discomfort [39]. For patients with neurodegenerative diseases such as AD that require long-term management, this method of administration not only improves patient compliance but also significantly reduces medical costs and improves quality of life due to its home self-management characteristics [39].

Moreover, it is important that the nasal delivery system can significantly reduce the risk of systemic adverse reactions. Traditional administration often requires extremely high doses to overcome BBB, leading to severe systemic toxicity. In contrast, nasal delivery, with its advantage of direct access to the target area, can maintain effective therapeutic concentrations while reducing the dosage, thereby minimizing the impact on non-target organs [40]. For example, research [41] shows that siRNA administered nasally can precisely inhibit the production of amyloid β-protein, delaying the progression of AD without causing significant systemic toxicity. Additionally, it is worth emphasizing that this route successfully avoids the first-pass effect in the liver, further optimizing the bioavailability of the drug.

However, it must be pointed out that there are still critical technical bottlenecks in nasal delivery. The first and foremost is the limiting effect of the nasal mucosal barrier: the tight junctions of the epithelial cells and the rich mucus layer create significant absorption barriers for macromolecular drugs such as siRNA [41]. At the same time, the mucociliary clearance mechanism (MCC) significantly shortens the drug residence time, severely affecting the administration efficiency [42]. Another core challenge lies in dose-limiting stimulation: excessive drug loading may damage the nasal mucosal epithelium, triggering local inflammatory responses [43], which not only affects treatment continuity but may also reduce patient acceptance [44]. Therefore, developing efficient trans-mucosal delivery carriers and precise dosage control schemes has become the key path to breaking through the current technical bottlenecks.

In summary, although nasal delivery has shown unique advantages in the treatment of CNS diseases, it still requires collaborative breakthroughs through innovative formulation technologies and delivery strategies to overcome existing limitations and realize its clinical transformation potential.

## 4. siRNA Nanocarrier Design Strategies

### 4.1. Basic Functions and Design Principles of Nanocarriers

The application of nanocarriers in siRNA delivery systems must adhere to core design principles to ensure their effectiveness and safety [45]. The primary goal is to protect siRNA from nuclease degradation: siRNA is easily degraded by nucleases in the body, thereby reducing its therapeutic efficacy [46]. To address this challenge, carrier systems such as lipid nanoparticles (LNPs), polymer nanoparticles, and inorganic nanoparticles encapsulate siRNA to form protective structures, significantly enhancing its stability [41,46,47,48]. For example, polymer-modified lipid nanocarriers (such as solid lipid–polymer hybrid nanocarriers) can effectively shield against enzymatic degradation and extend circulation time in vivo, thereby optimizing siRNA delivery efficiency [46]. Another key design principle is to optimize cellular uptake efficiency. The physicochemical properties of nanocarriers (such as nanoparticle size, surface charge, and hydrophobicity) directly affect the endocytosis effect [49,50,51]. For instance, studies have shown that surface charge (such as cationic modification) can significantly enhance cellular uptake efficiency, as charge variation can modulate the electrostatic interactions between the carrier and the cell membrane [49,51]. Meanwhile, surface functionalization strategies (such as coupling with arginine or cell-penetrating peptides) can significantly enhance the interaction between the carrier and the cell membrane by directly promoting membrane penetration or increasing affinity, thereby optimizing the uptake process [50,52,53]. Specifically, nanocarriers with a surface density of arginine and an arginine–arginine distance of less than 3 nm (with a size of about 10 nm) can achieve efficient non-endocytic uptake, avoiding the limitations of endocytic pathways [50]. In addition, the nanoparticle size and surface modifications (such as polyethylene glycol coating) have also been shown to affect protein corona formation, thereby indirectly regulating cellular uptake kinetics [51]. In achieving the ultimate goal of efficient siRNA therapy, targeted delivery and controlled release are key directions. By modifying targeting ligands (such as tumor cell surface receptor-specific ligands), nanocarriers can precisely deliver siRNA to target tissues, significantly enhancing therapeutic selectivity [41,54]. For example, biomimetic cell membrane coating technology can achieve specific tissue targeting through receptor binding mechanisms of tumor cell membranes [54], while PD-L1 siRNA nanocarrier systems enhance delivery efficiency in tumor models through surface ligand design [41]. At the same time, responsive release mechanisms (such as pH or temperature-responsive carriers) can trigger siRNA release under specific physiological conditions, which not only enhances efficacy but also reduces systemic side effects [47,48]. Specifically, ATP-responsive LNPs can be triggered to release by intracellular ATP in the tumor microenvironment, optimizing drug kinetics [47], while photo-crosslinked biodegradable polymer nanoparticles can achieve pH or enzyme-responsive dissociation under in vivo and in vitro conditions, promoting controlled release and reducing off-target effects [48]. Furthermore, successful endosomal escape is crucial for siRNA function, as siRNA must be released from endosomes into the cytoplasm to activate the RNA interference mechanism [48,55]. Research indicates that the design of carriers must integrate endosomal escape optimization (such as introducing polymers that promote membrane fusion or buffering capacity) to avoid nucleic acid degradation and ensure efficient gene silencing [55], which has been validated in lipid and polymer nanocarriers by adding intracellular release auxiliary components (such as tertiary amines) [48].

In summary, an efficient siRNA nano-delivery system needs to achieve the following in synergy: nucleic acid protection, efficient cellular uptake, targeted delivery, and controlled release, providing a comprehensive solution for gene therapy.

### 4.2. The General Types of Nanocarriers

In modern drug delivery systems, several classes of nanocarriers are widely studied for siRNA administration, including liposomes, polymeric nanoparticles, nanomicelles, and hybrid carriers.

Liposomes are composed of phospholipid bilayers, which can efficiently encapsulate water-soluble drugs and achieve rapid release [56]. Their main advantages lie in excellent biocompatibility and low toxicity, making them suitable for the delivery of antibiotics and antitumor agents [57]. However, they generally suffer from poor stability and are prone to degradation in vivo, which limits their long-term applications [58].

Polymeric nanoparticles are attractive due to their tunable physicochemical properties. By adjusting polymer type, molecular weight, and surface modification, drug release kinetics and targeting ability can be precisely regulated [59]. These carriers can effectively protect siRNA from enzymatic degradation, and, owing to their good biocompatibility, have broad potential in gene therapy and oncology [60]. Nevertheless, challenges remain regarding complex synthesis and unpredictable in vivo biodistribution that may reduce delivery efficiency [61].

Nanomicelles, formed by the self-assembly of surfactants or amphiphilic polymers, are particularly suitable for the delivery of hydrophobic agents [62]. They offer high drug-loading capacity and improved bioavailability [63]. For instance, micelle-based siRNA carriers have shown promise in enhancing local drug concentration in targeted tumor therapy [64]. Their limited stability and uncontrolled release behavior, however, are major barriers to clinical translation.

Hybrid carriers, such as solid lipid–polymer hybrid nanoparticles (SLPHNs), combine the structural advantages of lipids and polymers. Lipids provide enhanced stability and biocompatibility, while polymers allow fine-tuned release profiles and functional surface modifications [65]. Studies confirm that SLPHNs not only achieve efficient siRNA protection and delivery but also maintain low cytotoxicity, representing a promising next-generation platform that balances efficiency and safety.

In summary, each type of carrier offers distinct advantages and limitations: liposomes are biocompatible but unstable, polymeric nanoparticles excel in protection but are complex to prepare, micelles improve hydrophobic drug solubility but face release issues, and hybrid systems show synergistic properties that may overcome current bottlenecks.

### 4.3. Dendrimers as Specialized Carriers

Among the various nanocarriers investigated for siRNA delivery, dendrimers have attracted particular attention because of their unique structural features that are especially relevant to intranasal administration. Their generation-defined, highly branched architecture provides precise size control and abundant surface groups, allowing dense ligand conjugation and efficient siRNA binding. These characteristics help dendrimers address several challenges in nose-to-brain delivery, such as penetrating the mucus layer, crossing the nasal epithelium, and facilitating neuronal uptake [66,67,68,69,70,71,72].

Recent studies on polyamidoamine (PAMAM) dendrimers further highlight that surface functionalization with amino, carboxyl, polyvinyl alcohol chains, or fluorinated groups can significantly improve cellular uptake while reducing cytotoxicity, thereby enhancing biocompatibility [73,74,75,76]. Importantly, these modifications have been reported to facilitate mucosal penetration and prolong retention in the nasal cavity, which are critical factors for efficient nose-to-brain delivery. The structural generation of PAMAM also plays a crucial role; for instance, fourth-generation PAMAM with larger cavities and more surface groups has demonstrated higher siRNA loading capacity and controllable release behavior [77,78]. Stimuli-responsive modifications, such as pH- or temperature-sensitive linkers, enable targeted and timely release of siRNA in endosomal/lysosomal compartments after intranasal uptake, thereby improving therapeutic precision while minimizing systemic toxicity [79,80,81].

In vivo evidence also supports the potential of dendrimer-based carriers in nasal delivery systems. Kim et al. demonstrated that PAMAM-derived carriers enabled intranasal delivery of HMGB1 siRNA, resulting in target gene knockdown and neuroprotection in a post-ischemic brain model [82]. In addition, beyond nucleic acids, PAMAM dendrimers have also been shown to improve brain targeting of small-molecule drugs after intranasal administration, as illustrated by enhanced brain distribution of haloperidol [83]. Importantly, these findings confirm that dendrimers hold strong potential for CNS therapy by achieving efficient brain accumulation and functional improvement. Importantly, these carriers improved behavioral outcomes in disease models such as Alzheimer’s disease, indicating not only efficient brain distribution but also functional recovery [58,84].

Safety assessments have consistently demonstrated favorable biodistribution and low systemic toxicity of PAMAM after intranasal administration, with no major organ damage observed within therapeutic dose ranges [76]. These findings support their promise as safe and efficient nanocarriers for CNS-targeted siRNA delivery.

When compared with other nanocarriers, dendrimers offer more precise structural control and greater multivalency, allowing multiple functional groups—such as targeting ligands, protective coatings, or stimuli-responsive elements—to be incorporated into a single nanosystem. Liposomes and polymeric nanoparticles remain more widely reported, largely due to their established safety profiles and easier large-scale preparation [56,57,58,59,60,61]. However, these carriers generally provide less flexibility for fine-tuning particle architecture and surface chemistry. Hybrid systems combine stability with functional versatility, yet dendrimers still stand out for their high siRNA complexation capacity and customizable surface modifications. Taken together, dendrimers represent a structurally versatile and functionally adaptable platform with distinctive advantages that complement the strengths of other carrier systems.

## 5. Delivery Mechanism of siRNA Nanocarriers for Nasal Delivery

### 5.1. The Pathway Through the Nasal Mucosa

The study of nasal delivery of siRNA nanocarrier systems for the prevention and treatment of CNS diseases highlights the crucial delivery pathway across the nasal mucosa. Research shows that nasal administration enables direct delivery to brain tissue via the olfactory and trigeminal pathways, overcoming the core bottleneck of traditional administration [31].

First, the mechanism of direct access to brain tissue via the olfactory nerve pathway has been widely confirmed. The olfactory neurons in the nasal cavity have direct anatomical connections with the brain, allowing drugs to be rapidly transported into the brain through axons [45]. Experiments show that siRNA administered intranasally can efficiently reach the brain through this pathway [9]. The core advantage of this pathway lies in its rapid targeting characteristics, whereby drugs can directly reach the CNS target area and significantly enhance therapeutic effects [8]. For example, lipophilic C16-siRNA not only demonstrates excellent biocompatibility in Alzheimer’s disease models but also achieves long-lasting gene silencing, fully validating the potential of nasal administration [30].

Secondly, the trigeminal nerve pathway is also crucial as a key route for drug delivery to areas such as the brain stem. The branches of the trigeminal nerve are widely distributed in the nasal mucosa, providing a second direct pathway to the CNS for drugs. It is noteworthy that this pathway is particularly important for diseases involving pain perception, characterized by (i) significantly increased drug bioavailability, (ii) reduced side effects from systemic exposure, and (iii) lesion-specific delivery [9]. By designing targeted nanocarriers (such as surface-modified neuroaffinity peptides), the delivery efficiency of this pathway can be further enhanced [85].

Beyond these entry routes, accumulating evidence indicates that intranasally administered nanocarriers can redistribute from the initial olfactory and trigeminal targets to wider brain regions within minutes to hours. Studies suggest that glymphatic flow and perivascular transport may drive redistribution along interstitial and vascular pathways toward the cortex and hippocampus [86,87]. In addition, axonal transport and trans-synaptic relay can propagate carriers from primary olfactory or trigeminal neurons to higher-order regions [58,64]. Finally, CSF circulation allows carriers entering the subarachnoid space to spread across ventricular and cortical surfaces [88].

In conclusion, the dual olfactory and trigeminal nerve pathways provide a novel strategy for siRNA nanocarriers to achieve effective brain targeting. In-depth exploration of these mechanisms not only advances research on CNS disease therapies but also lays a critical theoretical foundation for clinical translation. With continued optimization of delivery system design (e.g., development of stimuli-responsive carriers), more efficient and targeted therapeutics are anticipated to emerge in the future.

### 5.2. Interaction Between Nanocarriers and Nasal Mucosa

Nanocarriers play an important role in nasal delivery, and their surface charge, nanoparticle size, and hydrophilicity are key factors affecting mucosal penetration [89]. First, the surface charge directly regulates the electrostatic interaction between the carrier and the nasal mucosa. Positively charged nanocarriers can enhance adhesion due to the negatively charged mucosal surface through charge attraction, significantly improving drug delivery efficiency [90]. For example, cationically modified nanoparticles exhibit stronger mucosal adhesion and cellular uptake capabilities, effectively promoting drug delivery [91]. Secondly, nanoparticle size significantly affects penetration efficiency. Nanoparticles with a diameter of 100–200 nm are more likely to penetrate the mucosal barrier, as their smaller size facilitates passage through intercellular gaps; larger particles are more likely to be retained by the mucosa, leading to decreased delivery efficiency [68]. Research has confirmed that optimizing the size of nanocarriers for nasal administration can significantly increase drug concentration in the brain, improving bioavailability and efficacy [92]. Additionally, hydrophilicity is another core parameter. Highly hydrophilic carriers can enhance mucosal adhesion through hydrogen bonding, forming a stable film on the mucosal surface and prolonging retention time [93]. It is noteworthy that prolonged retention time not only increases local drug concentration but also promotes trans-mucosal permeation. A typical example is hydrophilic nanoparticles exhibiting excellent adhesion and permeability in the nasal cavity, which is crucial for brain delivery systems [94]. It is important to note that mucosal retention time directly affects drug delivery efficiency. A longer retention time can significantly enhance local drug concentration in the nasal cavity, increasing bioactivity and therapeutic effects [95]. Surface modification (such as introducing hydrophilic/hydrophobic groups) can actively regulate retention characteristics: for example, functionalized nanoparticles can extend mucosal retention time by 40%, simultaneously increasing brain drug concentration and bioavailability [96]. It should be emphasized that retention time is closely related to the physicochemical properties of the carrier: larger particle sizes may extend retention but could sacrifice penetration efficiency, necessitating a balance between the two [93]. At the same time, drug release kinetics also play a key role—sustained-release carriers can maintain effective drug concentration in the nasal cavity, enhancing efficacy by prolonging action time [68].

In summary, the three key parameters of nanocarriers—surface charge, particle size, and hydrophilicity—along with mucosal retention time, collectively constitute the core governing factors for nasal delivery efficiency. Systematic optimization of these parameters provides a novel strategy for developing high-performance nasal drug delivery systems.

### 5.3. siRNA Release and Intracellular Delivery Mechanism

In the delivery of siRNA, the release kinetics of siRNA from nanocarriers are critically important, directly influencing therapeutic efficacy [97]. Studies demonstrate that siRNA release is governed by a combination of factors, including the physicochemical properties of the carrier (e.g., charge and hydrophobicity), environmental conditions (e.g., pH and ionic strength), and carrier–siRNA interactions [97,98,99]. For instance, when using cationic nanocarriers, the release rate is highly dependent on pH, carrier concentration, and loading time: siRNA loading in low-pH environments significantly enhances release efficiency, offering a novel strategy for tumor-specific siRNA delivery targeting the acidic tumor microenvironment [100]. Furthermore, advanced carriers such as functionalized LNPs promote endogenous siRNA release through specific binding to tumor cell surface receptors [101]; additionally, certain LNP designs enable ATP-triggered intracellular siRNA release, thereby improving gene silencing efficiency [102]. In light of these advancements, optimizing siRNA release kinetics can refine nanocarrier design and amplify therapeutic potential.

**Figure 2:**
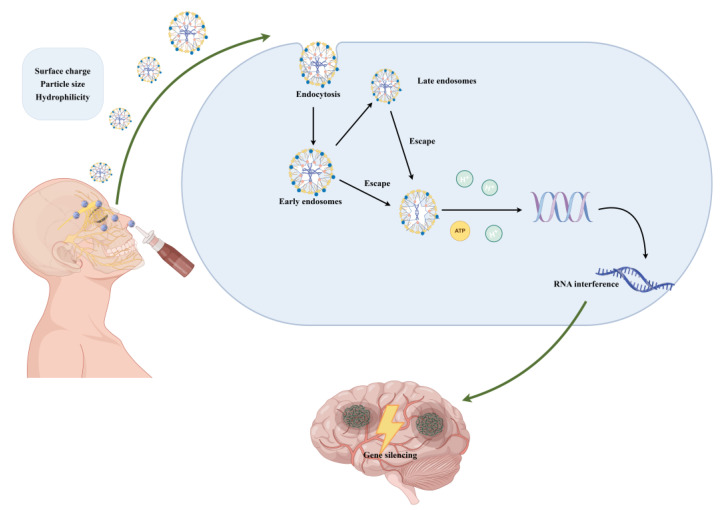
Intracellular trafficking and release mechanisms of siRNA nanocarriers. After uptake into target cells via endocytosis, siRNA-loaded nanocarriers are trafficked into endosomes. Effective delivery strategies enable endosomal escape, releasing siRNA into the cytoplasm where it binds to the RISC. This process leads to degradation of complementary target mRNA and subsequent gene silencing. Abbreviations: RISC, RNA-induced silencing complex; siRNA, small interfering RNA. [Created by Figdraw (www.figdraw.com, Accessed on: 12 September 2025), ID: SAPOOa6dd0].

Simultaneously, the cellular uptake pathways and intracellular transport mechanisms of siRNA represent another critical therapeutic bottleneck [103]. As illustrated in Figure 2, cells internalize siRNA-loaded nanocarriers via endocytosis, including classical pathways such as receptor-mediated and caveolae-mediated endocytosis, processes regulated by carrier properties [104]. Studies have demonstrated that intracellular trafficking mechanisms significantly impact siRNA bioavailability [105]. For instance, cell-penetrating peptide (CPP)-modified nanocarriers enhance uptake efficiency by binding to cell surface receptors or directly penetrating membrane structures [106]. Following endocytosis, siRNA is typically trafficked to the endoplasmic reticulum or lysosomes before being released into the cytosol via endosomal escape mechanisms to participate in RNA interference; photosensitive compound-based nanocarriers can induce endosomal membrane fusion under light irradiation, preventing siRNA degradation and facilitating release [107]. Therefore, elucidating these mechanisms provides a solid theoretical foundation for clinical applications.

## 6. Research Progress on In Vitro and In Vivo Delivery of siRNA Nanocarriers via Nasal Cavity

### 6.1. Delivery Efficiency and Gene Silencing Effect in Vitro Cell Models

When studying the delivery efficiency of siRNA, the choice of cell line is crucial [108]. Different cell lines exhibit significant differences in their response to siRNA carriers, which directly affects the assessment of transfection efficiency and gene silencing effects; for example, in in vitro tests, the uptake capacity and silencing efficiency of the carrier can vary significantly depending on the cell type when using microglia or dendritic cells [109,110]. Therefore, selecting an appropriate cell line not only allows for an accurate assessment of gene silencing effects but also provides key information for the transfection efficiency of the carrier [111]. In recent years, researchers have widely adopted various in vitro models for validation, including tumor-associated cell lines and immune cells, to comprehensively test the performance and biocompatibility of siRNA delivery systems [108,110]. In typical cases, siRNA delivery systems based on polymeric nanocarriers have demonstrated the ability to efficiently downregulate immune-related genes, such as targeting the CD40 gene in mouse models, significantly inhibiting the activation of macrophages and dendritic cells, thereby enhancing anti-tumor effects [110]. At the same time, using surface-modified nanocarriers (such as bola-type carriers modified with hyaluronic acid) to target specific cell surface markers (such as Bcl-2 related pathways) significantly improved transfection efficiency and silencing specificity, which has been confirmed in both in vitro and in vivo experiments to enhance cell-specific delivery capabilities [111]. These findings, based on systematic validation of the literature, emphasize the critical role of cell line selection strategies in optimizing siRNA therapy.

In terms of carrier types, LNPs and polymer nanoparticles are widely used [65,112], with the former favored for its excellent biocompatibility and high transfection efficiency [112]. Specifically, liposomes excel in cell membrane penetration and intracellular siRNA release, effectively maintaining siRNA activity to enhance gene silencing [112]. Additionally, metal–organic frameworks (MOF) nanoparticles also show potential in specific cell types, capable of overcoming intracellular barriers to improve delivery efficiency [98].

It is worth noting that, in addition to transfection efficiency, the impact of nanocarriers on cell viability is also a key evaluation indicator [46]. An ideal carrier needs to balance efficient delivery with low cytotoxicity; however, some polymer carriers may induce apoptosis and toxic reactions at high concentrations, leading to a significant decrease in viability [113]. In contrast, new hybrid carriers such as SLPHNs demonstrate significant advantages: studies have confirmed that they not only achieve efficient siRNA delivery but also maintain cell viability and function across various cell lines [114]. This suggests that by optimizing the composition and structure of carriers, it is possible to enhance endocytosis efficiency while reducing toxicity, thereby improving gene silencing effects [46].

In summary, the optimization of siRNA nanocarriers needs to consider multiple factors such as cell line compatibility, transfection efficiency, and biosafety. Thanks to advancements in nanomaterial engineering, new carriers that combine efficient delivery with good biocompatibility will continue to emerge, opening new pathways for gene therapy in CNS diseases.

### 6.2. Nasal Delivery Efficacy and Targeting in Animal Models

Nasal delivery of siRNA technology can overcome the limitations of the BBB, achieving efficient delivery of siRNA in brain tissue [41]. Studies have shown that after nasal administration, siRNA can be widely distributed in the brains of mice and significantly silence target gene expression [39]. For example, in AD treatment studies, multifunctional nanocarriers (Rapa@DAK/siRNA) delivered BACE1 siRNA to the brain via the nasal route, significantly reducing BACE1 expression and decreasing β-amyloid (Aβ) deposition by activating the autophagy pathway, ultimately improving cognitive function in transgenic AD mice [39]. This result confirms the translational potential of nasal delivery of siRNA for treating CNS diseases.

In this technological system, dendritic polymers as novel nanocarriers exhibit unique advantages. They can efficiently encapsulate siRNA and deliver it directly to brain tissue via the nasal cavity, circumventing the limitations of the BBB [66]. Research shows that dendritic polymers (such as PAMAM) demonstrate excellent biocompatibility when delivering small or large molecule drugs; for example, mouse models confirm that PAMAM carriers can accurately deliver drugs to the brain without systemic toxic reactions [9]. This targeted delivery strategy provides innovative ideas for enhancing drug bioavailability.

Further research needs to systematically evaluate efficacy and safety. In terms of efficacy, behavioral tests (such as spatial learning and memory), neurobiochemical indicators (Aβ levels, autophagy protein expression), and brain imaging techniques can be used to comprehensively analyze the distribution characteristics of siRNA, targeting gene silencing effects and the degree of pathological improvement [58,115]. As previously mentioned, the study employed various cognitive behavioral experiments to assess the recovery of neural function in AD mice [58]. Safety surveillance should cover indicators such as weight changes, organ function (liver and renal function), and histopathology (nasal mucosa, inflammatory infiltration in brain tissue) to clarify the biotoxicity of the carrier and siRNA [58,116]. These standardized evaluation systems lay a scientific foundation for the clinical translation of nasal delivery of siRNA.

In addition to the individual studies discussed above, representative examples of siRNA nanocarrier delivery in both in vitro and in vivo models are summarized in Table 1, highlighting the siRNA targets, models employed, and major therapeutic outcomes.

### 6.3. Challenges and Prospects of Preclinical Research

In the preclinical study of the nasal delivery siRNA nanoparticle system, the main challenges focus on three aspects: large-scale production and quality control, long-term safety and immune response assessment, and optimization of the delivery system itself [58].

Large-scale production and quality control constitute the primary challenge. Although many nanoparticles have demonstrated excellent siRNA delivery capabilities under small-scale laboratory conditions, efficiently, economically, and reproducibly scaling up these systems to meet clinical demands remains a daunting task [56,66]. This requires the development of robust manufacturing processes to ensure high consistency and reliability in the physicochemical properties (such as particle size distribution, surface charge, morphological characteristics, etc.) and biological performance (such as drug loading capacity, release kinetics) of nanoparticles between different production batches [76]. Furthermore, strict quality control is indispensable, especially the precise monitoring of the aforementioned key parameters to avoid potential poor efficacy or safety risks in clinical applications [58].

Long-term safety and immune response assessment are equally crucial. The biocompatibility of nanocarriers in vivo and their potential to induce immune responses are core aspects that must be thoroughly evaluated in preclinical studies [117]. Although some studies have shown that certain carriers have lower immunogenicity, long-term animal experiments are still needed to systematically assess their potential chronic effects and toxic side effects on the immune system [118]. It is noteworthy that in the context of cancer treatment or other chronic diseases requiring long-term medication, detailed safety data are of decisive significance for the design and ethical approval of subsequent clinical trials [119]. Therefore, future research should focus on developing delivery systems that combine high efficacy and high safety, minimizing the risk of adverse drug reactions in clinical applications [120].

**Table 1:** Representative studies on siRNA nanocarrier delivery in vitro and in vivo models.

siRNA Target/Strategy	Model (In Vitro/In Vivo)	Key Findings/Results	Reference
CD40 siRNA (polymeric nanocarriers)	Mouse immune-related models (in vitro/in vivo, macrophages and dendritic cells)	Downregulated CD40; inhibited macrophage and dendritic cell activation; enhanced anti-tumor effects	[112]
Bcl-2 pathway siRNA (HA-modified bola-type carriers)	Tumor-associated cell lines (in vitro/in vivo)	Improved transfection efficiency and silencing specificity; enhanced cell-specific delivery	[113]
Generic siRNA (liposomes)	Multiple in vitro cell lines	High biocompatibility; efficient membrane penetration; maintained siRNA activity	[34]
Generic siRNA (MOF nanoparticles)	Specific cell types (in vitro)	Overcame intracellular barriers; improved delivery efficiency	[40]
Generic siRNA (solid lipid–polymer hybrid nanoparticles, SLPHNs)	Various in vitro cell lines	Efficient siRNA delivery; maintained cell viability; reduced cytotoxicity	[46]
BACE1 siRNA (Rapa@DAK/siRNA nanoparticles)	Alzheimer’s disease transgenic mice (in vivo, nasal delivery)	Reduced BACE1 expression; decreased Aβ deposition; improved cognition	[58]
PAMAM dendrimers (generic siRNA delivery)	Mouse models (in vivo, nasal delivery)	Accurate brain delivery; favorable biocompatibility; no systemic toxicity	[83]
HMGB1 siRNA (PAMAM dendrimers)	Post-ischemic brain injury mice (in vivo, intranasal)	Target knockdown; robust neuroprotection	[82]
ApoE siRNA	Alzheimer’s disease mouse model (in vivo)	Reduced amyloid burden; improved neuronal survival	[119]
NMDAR siRNA (RVG-exosomes)	Orofacial neuropathic pain rats (in vivo)	Suppressed central sensitization; alleviated pain	[22]

To address these challenges, researchers are actively exploring innovative solutions. For example, designing biomimetic nanocarriers or developing multifunctional nanoparticles aims to enhance the stability and targeting specificity of siRNA, thereby reducing adverse effects on non-target tissues [121]. At the same time, using Artificial Intelligence (AI) to assist in optimizing the structural and functional parameters of nanocarriers also provides new ideas for improving delivery efficiency and safety [122].

In summary, although preclinical studies on nasal delivery of siRNA nanocarriers have made certain progress, achieving clinical translation and widespread application in the prevention and treatment of CNS diseases still requires overcoming bottlenecks in large-scale production, establishing strict quality control standards, and conducting in-depth long-term safety assessments. Future research should focus on addressing these key issues to accelerate the transition of this technology from the laboratory to clinical practice.

## 7. Challenges and Future Directions of Nasal Delivery siRNA Nanocarrier Systems

### 7.1. Technology and Biological Barriers

The nasal delivery system provides important prospects for the treatment of CNS diseases, especially because it can directly deliver drugs to the CNS through its anatomical connection with the brain through the anatomical connection between the nasal cavity and the brain [31]. However, the nasal mucosa, as a powerful biological barrier, poses significant challenges to drug delivery due to its protective functions and clearance mechanisms, especially when delivering siRNA-based nanocarriers [41].

To overcome these obstacles, researchers have adopted various strategies. Modified nanocarriers (such as modified dendritic polymers) can enhance adsorption on the nasal mucosa and reduce clearance rates [9]. In addition, multifunctional nanoparticles (for example, particles loaded with Aleuria aurantia lectin (AAL) ligands) can specifically bind to L-fucose in the olfactory epithelium, significantly improving brain delivery efficiency [39]. These optimization measures effectively enhance the bioavailability of siRNA and reduce nasal clearance rates, thereby enhancing its therapeutic efficacy.

It is worth noting that the biodegradability and long-term safety of the carriers are core considerations for this delivery system [123]. An ideal nanocarrier should possess good biocompatibility and biodegradability to ensure safe metabolism after drug release, avoiding cumulative toxicity [123]. For example, polymer-based carriers (such as dendritic polymers) can gradually degrade in the body through hydrolysis or enzymatic degradation, promoting drug release and reducing tissue retention time, thereby lowering potential toxicity [9]. Good degradation characteristics also help improve clinical acceptance.

Nevertheless, even though many carriers show good biocompatibility, in-depth long-term safety assessments are still required, especially under repeated dosing conditions. It is crucial to evaluate toxicity and side effects in different disease models (such as AD models). Existing studies have demonstrated [39] that modified nanocarriers delivered via the nasal cavity can effectively reduce amyloid β-protein deposition and improve cognitive function, preliminarily confirming their therapeutic potential and safety.

In summary, the nasal delivery siRNA nanocarrier system has made significant progress in overcoming biological barriers, but the efficacy and safety of its clinical applications still need to be ultimately established through in-depth research on the biodegradability of the carriers and long-term safety.

### 7.2. Strategies for Improving Delivery Efficiency and Targeting

In siRNA therapy, delivery efficiency and targeting are core factors determining efficacy [50,66]. To enhance delivery efficiency, multifunctional modified nanocarriers have gradually become the focus of research: by modifying targeting ligands on the surface of the carriers, precise targeting of specific cells or tissues can be achieved [39]. For example, nanocarriers designed for AD utilize the aforementioned ligands to deliver BACE1 siRNA, which can effectively cross the BBB after intranasal administration, significantly improving cognitive function in AD model mice [39]. Similarly, targeting modifications based on tumor microenvironment markers (such as CD44) can enhance tumor cell uptake of siRNA, thereby improving efficacy [124].

It is worth noting that multifunctional modifications can not only optimize targeting but also reduce the non-specific distribution and toxicity of siRNA [124]. Taking hyaluronic acid (HA)-modified cationic polyurethane nanoparticles as an example, they significantly enhance the uptake efficiency of siRNA by MCF-7 cells through CD44-mediated endocytosis and effectively inhibit STAT3 expression [124], while simultaneously improving the safety and efficacy of the treatment.

In recent years, combination delivery strategies have attracted attention due to their synergistic effects—co-delivering siRNA with chemotherapy drugs (such as Doxorubicin) or immunotherapeutics on the same nanocarrier [125]. For example, redox-sensitive “2-in-1” nanoparticles can selectively release siRNA and Doxorubicin in the tumor microenvironment, synergistically inhibiting tumor growth [125]. Further studies have shown that biocompatible carriers (such as pH-responsive polymer nanoparticles) co-delivering siRNA with chemotherapy drugs (such as Hydroxycamptothecin hydrochloride) can not only improve drug bioavailability and reduce toxic side effects but also achieve superior anti-tumor effects compared to single agents through spatiotemporal synchronous release [126].

The key to successfully implementing such strategies lies in the rational design of carriers: it is necessary to comprehensively consider drug release kinetics, cellular uptake pathways, and microenvironment characteristics. Overall, multifunctional modifications and combined delivery provide new directions for the clinical translation of siRNA, with significant potential in the future.

### 7.3. Key Issues in Clinical Translation

In the clinical translation of siRNA nanoparticle carrier systems, the systematic evaluation of pharmacokinetics (PK) and pharmacodynamics for CNS diseases is crucial [85]. Pharmacokinetics needs to comprehensively assess the absorption, distribution, metabolism, and excretion (ADME) characteristics of the drug [85]. Studies have shown that siRNA nanoparticles can effectively penetrate the BBB, significantly enhancing the precision of drug delivery and the controllability of biodistribution, thereby improving curative effects [64]. However, insufficient stability of the carrier in vivo and limited targeting leading to non-specific distribution remain current challenges [85]. In addition, pharmacodynamic evaluation needs to systematically analyze the inhibitory effects of siRNA on target gene expression and the impact on related pathways to clarify therapeutic potential and mechanisms, providing key efficacy and safety baseline data for clinical trial design [85].

Furthermore, with the development of gene therapy technology, the clinical translation of such systems faces strict laws and regulations, as well as ethics reviews [127]. Regulatory agencies in the US and Europe (such as FDA/EMA) require the provision of non-clinical and clinical data covering safety, toxicity, and efficacy [127]. Research must adhere to the principle of informed consent, fully informing participants of the risks and benefits while ensuring patient privacy and data compliance [127]. In this process, researchers need to collaborate with the ethics committee to ensure protocol compliance, laying the foundation for the translation pathway [127].

Ultimately, the rationality of clinical trial design directly affects the success rate of translation. The study needs to clarify the trial objectives, protocols, and endpoint indicators, especially focusing on patient compliance issues—which significantly impact the success rate of long-term complex dosing regimens [84]. Optimization strategies include simplifying the administration method, improving carrier characteristics to enhance patient tolerance, and verifying the reliability of results through scientific statistical methods [84]. Through the above measures, the clinical application of siRNA nanocarriers in the treatment of CNS diseases can be accelerated.

## 8. Conclusions

With the continuous advancement of biomedical technology, nasal delivery siRNA nanocarrier systems have shown great potential for achieving targeted therapy for CNS diseases through a non-invasive route. This innovative approach not only provides new ideas for the treatment of complex diseases such as neurodegenerative diseases and brain tumors but also offers effective solutions to the limitations of traditional treatment methods.

Dendritic polymers and other nanocarriers show significant advantages in improving the stability, delivery efficiency, and safety of siRNA. By optimizing the design of these carriers, we can greatly enhance the bioavailability of siRNA and ensure its effective release in target tissues. The diversity and flexibility of these nanocarriers allow researchers to design targeted solutions based on different therapeutic needs. However, despite existing studies indicating that nasal delivery can achieve efficient brain siRNA delivery and reduce systemic side effects, attention must be paid to the differences between studies, especially since results may vary significantly under different models and experimental conditions. Therefore, future research should focus on standardizing experimental methods to form more consistent conclusions.

Further research should focus on optimizing carrier design, gaining a deeper understanding of delivery mechanisms, and improving safety evaluation during the clinical translation process. Currently, studies on the release mechanisms and intracellular transport pathways of siRNA are still insufficient, which limits our comprehensive understanding of the in vivo behavior of nanocarriers. Future research should consider employing advanced imaging techniques and molecular biology methods to reveal the dynamic distribution and action mechanisms of siRNA in vivo, thereby providing a more solid foundation for clinical applications.

In addition, interdisciplinary collaboration will be key to advancing the clinical applications of nasal delivery siRNA nanoparticle systems. Experts from fields such as biomedical engineering, medicinal chemistry, and neuroscience participating together can accelerate the research and optimization of technology. By integrating knowledge and techniques from different fields, we will be able to more effectively address complex biomedical challenges and promote the clinical translation of siRNA therapeutic strategies.

In summary, the nasal delivery siRNA nanoparticle system opens up new directions for the treatment of diseases of the CNS. Although there are still many challenges ahead, its vast potential for clinical applications cannot be ignored. With the deepening of research and advancements in technology, we look forward to continuous breakthroughs in this field, achieving broader clinical applications and ultimately bringing benefits to patients.

## Data Availability

No new data were created or analyzed in this study. Data sharing is not applicable to this article.
